# Allergic contact dermatitis to Compositae: An Australian case series

**DOI:** 10.1111/cod.14162

**Published:** 2022-06-07

**Authors:** Nisal Punchihewa, Amanda Palmer, Rosemary Nixon

**Affiliations:** ^1^ Occupational Dermatology Research and Education Centre Skin Health Institute Carlton Victoria Australia

**Keywords:** airborne contact dermatitis, Asteraceae, chronic actinic dermatitis, Compositae, gardening, hand dermatitis, mowing

## Abstract

**Background:**

Allergic contact dermatitis (ACD) to Compositae is caused by sensitisation to sesquiterpene lactones (SQLs) and subsequent exposure can occur from direct handling or from airborne transmission. Plants from the Compositae family are ubiquitous globally and their plant extracts are also used in various products.

**Objectives:**

Investigation of contact allergy (CA) and allergic contact dermatitis (ACD) to Compositae at a single dermatology centre.

**Methods:**

A retrospective case review was performed on patients undergoing patch testing to Compositae between January 2011 and December 2020 in Melbourne, Australia.

**Results:**

Of 3679 patients, 44 (1.2%) patch tested positive to Compositae and 19 (43.2%) reactions were deemed relevant. Thirteen cases (68.4%) were from direct contact with Compositae plants, mostly in gardeners. Six cases (31.6%) were from personal products and all these patients were female. Involvement of the face was significant (*p* = 0.007). Simultaneous allergic reactions included SQL mix in eight (42.1%), fragrance mix in seven (36.8%), potassium dichromate in three (15.8%) and colophonium in two (10.5%) cases.

**Conclusion:**

Contact with Compositae from gardening contributed most cases of ACD; however, personal products accounted almost one‐third of cases. Treatment options remain limited and avoidance is the most important aspect of management.

## 1 INTRODUCTION

Allergic contact dermatitis (ACD) to the Compositae family of plants causes a familiar yet challenging clinical scenario for dermatologists. There are over 24 000 species of Compositae that are widespread in grasslands, woodlands and alpine habitats.^1^ The scientific name *Asteraceae* is derived from the Greek word ‘Aster’ which means star. It refers to the configuration of petals arranged radially around a central disc, in turn made up of smaller discs. What appears initially as a single flower is therefore a ‘composite’ of smaller flowers hence the older term ‘Compositae’.^2^

Weeds from this family are known to be aggressive and competitively take over vacant land because of their high reproductive capacity. South Australian dermatologist John Burry coined the term ‘Australian Bush Dermatitis’ in the 1970s to describe Compositae dermatitis in outdoor workers experiencing seasonal symptoms with improvement when spending time in the city.^3^ In America, ‘Ragweed Dermatitis’ was described among farmers from areas with *Ambrosia artemisiifolia*. In India, Feverfew (*Parthenium hysterophorus*) was introduced accidentally and resulted in large numbers of Compositae dermatitis cases.^4^, ^5^

The sensitizing component of Asteraceae is a group of biologically active metabolites called sesquiterpene lactones (SQLs).^6^ These lactones contain an α‐methylene‐γ‐lactone skeleton which is an immunochemical requisite for Compositae dermatitis.^7^ The lactone ring has been shown to chemically react and form covalent bonds with nucleophilic residues of proteins in the skin, resulting in the immune reaction that causes ACD.^8^

Compositae dermatitis typically involves exposed areas of the body such as the face, neck, ‘V’ of the neck, hands and forearms.^9^ The risk of exposure is higher during summer due to increased circulation of dried plant matter^10^ and exposed skin in hot weather. Acute vesicular reactions can occur particularly during summer months when flares are common. Repeated exposure eventuates in the typical presentation of chronic and lichenified skin as seen in atopic dermatitis.^3^

Although the typical clinical history of Compositae dermatitis involves a middle‐aged man with direct exposure to Compositae,^11^ exposure can be from various products containing the plant extract.^12^, ^13^ These products may range from ingested herbal medications including teas for muscle cramps to cosmetics, such as eye creams.^14^ Patients can also develop CA at any age but childhood atopic eczema and allergic rhinitis have been shown to be risk factors.^15^

Compositae dermatitis may be complicated by chronic actinic dermatitis (CAD) which is associated with photosensitivity.^16^ A ‘Compositae dermatitis syndrome’ has been described in previous literature and alludes to the challenge of distinguishing between these two variants.^17^ CAD appears similar clinically but classically spares photo protected sites including the upper eyelids, submandibular and retorauricular skin. In Compositae dermatitis, these sites are involved due to airborne exposure.^18^ The severity of both forms can range from localized dermatitis on the hands and face to widespread involvement. This may relate to different sources of exposure, cross‐sensitisation to similar allergens and endogenous photosensitivity.^15^

## 2 METHODS

We conducted a retrospective review of 3679 patients who underwent patch testing to Compositae at our tertiary dermatology centre in Melbourne, Australia between 2011 and 2020. Allergens were obtained from Chemotechnique Diagnostics (Vellinge, Sweden) and were secured on to the skin with Finn Chambers or Allergeaze test chambers (SmartPractice) for 48 h. Patch test readings were performed according to the International Contact Dermatitis Research Group (ICDRG) guidelines on Day 2 (D2) and Day 4 (D4).

All patients were tested with the Australian Baseline Series ABS‐1000 (Chemotechnique Diagnostics) and additional series were selected relevant to each patient presentation. The ABS‐1000 series includes both SQL Mix (Costunolide, Alantolactone, Dehydrocostus lactone) and Compositae Mix II (*Anthemis nobilis*, *Chamomilla recutita*, *Achillea millefolium* extract, *Tanacetum vulgare* extract, *Arnica montana* extract, Parthenolide). Plant extracts brought in by patients were prepared by separating into components (flower, leaf or stem) and mixing with acetone.

The chi‐square test was used to determine statistical significance between categorical variables and a *p* value of 0.05 was considered significant. Statistical analysis was conducted using SPSS for Macintosh (version 23.0; IBM Corp.).

## 3 RESULTS

Of 3679 patients that underwent patch testing to Compositae, 44 (1.2%) reacted (Table 1). Among the group that reacted, 19 (43.2%) had a relevant reaction and further details for each case are provided in Table 2. The mean age of patients with relevant reactions was 54 years.

**TABLE 1:** MOAHLFA (male, occupational dermatitis, atopic dermatitis, hand, face, leg and age >40) characteristics in for patients with positive patch tests for Compositae at our institution between January 2011 and December 2020

Index	Positive reactions (percentage of total sensitized) *n* = 44	Non‐sensitized patients (percentage of total non‐sensitized) *n* = 3617	*p* Value using chi‐squared test
Male	15 (34%)	1027 (28%)	0.41
Occupational dermatitis	4 (9%)	563 (16%)	0.24
Atopic dermatitis	10 (23%)	1304 (36%)	0.07
Hand dermatitis	16 (36%)	1072 (30%)	0.33
Leg dermatitis	1 (2%)	235 (6%)	0.26
Face dermatitis	19 (43%)	917 (25%)	0.007*
Age above 40	31 (70%)	2044 (57%)	0.84

*Note*: * Significance with *p* < 0.05.

**TABLE 2:** Patients with relevant reactions to Compositae at our tertiary dermatology centre between January 2011 and December 2020

Patient	Age (years)	Sex	Occupation	Exposure to Compositae	Medical history	Presentation	D2 reaction	D4 reaction	Other reactions
1	79	M	Retired banker	Gardening	Asthma Eczema Hay fever Emphysema AMI HTN	Eruption over the forehead, back of neck and vertex of scalp settling over winter	+	+	Colophonium Fragrance mix SQL
2	59	M	Retired abalone diver	Hunting	Asthma Eczema	Dermatitis of face, neck and hands	++	++	Fragrance mix SQL
3	58	M	Construction	Gardening	Hearing impairment	Erythematous to violaceous scaly plaques on hands and bilateral elbows. Mild confluent rash on anterior and posterior neck. Lichenified eczematous changes on the lower shins bilaterally	−	+	Fragrance mix Potassium dichromate SQL
4	61	M	Management	Gardening	Asthma Hay fever	Dermatitis over the face, chest and forearms. Mild dermatitis and erythema over back	−	+	
5	50	F	Unknown	Gardening	Nasal polyps	Facial dermatitis and intermittent eczematous eruptions over hands	−	+	Fragrance mix
6	37	F	Office worker	Gardening	Smoker	Hand and face dermatitis	+/−	+	*Myroxylon pereirae* (Balsam of Peru)
7	56	F	Nun	Helped in the process of making up cosmetics and toiletries in the monastery		Dermatitis over the forehead near the hair line and over the back at the waistline	−	+	Colophonium Potassium dichromate
8	36	F	Teacher	Renergie day cream contained sunflower		Facial dermatitis with occasionally weepy skin	−	+	Cobalt Nickel
9	67	M	Retired gardener	Gardening		Dermatitis hands and elsewhere including back and limbs. Also patchy discoid eczema on back	+/−	+	Fragrance mix SQL
10	69	F	Unknown	Pain relief spray, contained *Arnica montana*		Dermatitis on face and back	−	+	
11	64	F	Computer analyst	Gardening	HTN	Persistent eyelid swelling and lymphoedema	+	+	Cobalt Nickel
12	29	F	Marketing	Personal cosmetic products		Papular rash to face around mouth then cheeks and forehead	−	+	
13	36	F	Office worker	Body wash containing *Helianthus annuus* seed oil		Facial and hand dermatitis worsening over lips and eyelids	−	+	
14	69	F	Social work	Gardening	Asthma	Hand dermatitis and occasional whole body dermatitis	++	++	Fragrance mix SQL
15	57	M	Gardener	Gardening		Eczematous rash on face and hands	+++	+++	Potassium dichromate Methylisothiazolinone SQL
16	33	M	Welder	Gardening		Mild patchy eczematous rash on the forearms and across forehead	−	++	
17	46	M	Gardener	Gardening		Dermatitis of dorsal palms and fingers with involvement of ventral wrist, forehead and posterior neck. Minor eruption on ankles at the top of socks	+++	++	*M. pereirae* (Balsam of Peru) SQL
18	58	M	Gardener	Gardening		Diffuse facial eczema involving eyelids and involving anterior and posterior neck. Scaly, vesicular changes on palms with marked dermatitis on bilateral feet and ankles	+	++	SQL
19	54	F	Music Teacher	Pain relief spray contained *A. montana*	Asthma Hay fever	Cheilitis	−	+	Fragrance mix

Three patients (15.8%) were gardeners by occupation while 12 (63.2%) had direct exposure to Compositae from gardening. Eight out of 12 (66.6%) who performed gardening were males. Six out of 19 (31.6%) cases with relevant reactions were exposed to Compositae through cosmetic products and a pain‐relief spray. These six cases were all female. A single patient was exposed to Compositae during hunting.

Only involvement of the face was significant when compared to patients that did not react to Compositae (*p* = 0.007). In 19 patients with relevant reactions, dermatitis involved combinations of the face, neck, chest, upper and the lower limbs. Patients 3, 9, 14, 17 and 18 had involvement of the lower limbs and these patients all had their exposure to Compositae from gardening.

Simultaneous reactions for SQL mix were observed in eight patients (42.1%), followed by fragrance mix (seven patients, 36.8%), potassium dichromate (three patients, 15.8%) and colophonium (two patients, 10.5%).

## 4 DISCUSSION

The majority of cases of ACD to Compositae occurred from gardening (63.2%) and 25% of those cases were occupational. Patients were mostly middle‐aged and eight of these 12 patients (66.6%) were male. Involvement of the face was significant in those who reacted to Compositae compared to those that did not. The hands and forearms were most commonly involved in gardeners, followed by the face and lower limbs. A seasonal component of severe flares during summer with remission in winter months was observed in Patients 1, 2 and 15. These patients were diagnosed with CAD exacerbated by ACD to Compositae, a phenomenon previously documented in the literature.^16^, ^17^

Patient 6, a gardener, also had probable airborne contact with Compositae from the daisies planted outside her bedroom window. Patient 14 was known to have direct contact by handling lettuce (*Lactuca sativa*)^1^ with bare hands from her vegetable garden. Patient 17 regularly removed wormwood (*Artemisia absinthium*)^1^ weeds without using protective gloves and had severe dermatitis of their hands and forearms. Patient 2, who was exposed to Compositae through the sport of hunting, had dermatitis of the face, neck and hands, which were all likely to be exposed during this activity.

Almost a half of all positive reactions to Compositae were relevant. Approximately half (52.6%) of the relevant reactions occurred in females and six (31.6%) were caused through the use of personal products. The pattern of distribution was consistent with the area of skin that the product was applied to (Table 2). Cosmetic products and a bodywash were among the products that led to Compositae dermatitis in our cohort (Table 2).

In patients with non‐relevant reactions where clinical information was available, dermatitis of the eyelids was prevalent. These patients did not have seasonal variations in their symptoms, had indoor occupations and were not found to be using personal products containing Compositae extracts.

### 4.1 Cross‐reactions

Simultaneous reactions for SQL mix were observed in eight patients (42.1%), fragrance mix in seven (36.8%), potassium dichromate in three (15.8%) and colophonium in two (10.5%) cases. The overlap of positive patch tests to Compositae and SQL mix is to be expected. SQL mix is included in our baseline series as it is more sensitive in conjunction with Compositae mix for detecting Compositae dermatitis.^19^

Fragrance mix was relevant through use of personal care products which may also contain SQL if the fragrance component was derived from a member of the Compositae family. The association between fragrance mix and Compositae has also been recognized in the literature.^20^, ^21^

Colophonium is found in cosmetics such as perfumes, mascara and eyeshadows as well as in adhesives.^12^, ^13^ Three out of four patients had relevant reactions through the use of Grippo (used as a tackifier in bowling), Red Tiger balm and Elastoplast tape. Oxidized derivatives of the diterpenoid abietic acid have been shown to be the most important allergen in colophony. It can be found in several other plant families including Asteraceae which may explain the cross‐reactivity observed.^22^, ^23^

Cross‐reaction of Compositae with potassium dichromate is not a well‐known association but this link may be explained by outdoor occupational practices. Patient 3, a retired construction worker who also did lawn mowing, had simultaneous reactions to both allergens. The patient previously worked with wet cement which likely caused sensitisation to potassium dichromate and had subsequent exposure from leather shoes.

### 4.2 Avoidance

Avoiding direct exposure to Compositae may be challenging given they are a ubiquitous weed globally. New mobile apps to detect plant species may help patients avoid exposure but further validation is required.^24^ Direct handling could also occur in the preparation of edible variants, most commonly lettuce and artichoke while leaves of chrysanthemum and marigold species are used as flavouring or in salads, although this was not observed in our case series.^1^

In our experience, patients from our state of Victoria experienced problematic exposure to flowering weeds such as capeweed *(A. calendula)* and dandelions *(Taraxacum officinale)*. Pollen itself does not contain SQLs but rather the circulation of plant matter during dry months has been suggested as the primary cause of airborne transmission.^10^ Capeweed is distributed across all states and territories of Australia and flowers between August and December.^1^ Most species of dandelion flowers in the warmer months of September to April.^1^ Activities such as lawn mowing further contribute to airborne exposure of dry plant matter.

Compositae extracts have been used in cosmetic products for many decades and have even been reported to cause ACD from its use in insect repellent.^3^, ^14^ Patient 10 developed ACD from their muscle pain spray containing extracts from *A. montana*, highlighting the use of Compositae beyond cosmetics. Patients allergic to Compositae should choose their personal products carefully.

Systemic contact dermatitis from ingestion of allergens such as nickel and cobalt has been well described in the literature but high‐quality evidence is lacking for Compositae.^25^ SQLs can be found in lettuce, chamomile tea and other herbal medicines and some reports describe a temporal relationship of dermatitis to oral challenges. A 35‐year‐old‐lady with recalcitrant Compositae dermatitis was found to have a diet consisting of food products from the Compositae family. She observed vast improvement in her skin condition within 2 weeks of excluding these foods from her diet.^26^ Although none of our patients had oral exposure to Compositae, this less common mode of exposure should also be considered when determining the relevance of positive patch tests.

## 5 MANAGEMENT

The management of Compositae dermatitis is challenging as removal of the allergen is the mainstay of treatment but is not always achievable. Simple measures include bathing after being outdoors, regularly washing clothes and eliminating Compositae weeds in household gardens. Compositae flowers kept indoors for decorative purposes (such as dandelion or chrysanthemum spp.) should be identified and replaced. A broad spectrum sunscreen is appropriate especially if photosensitivity is suspected.

In instances of occupational exposure such as in gardeners, the use of protective gloves, protective clothing and facial protection is recommended. Skin abrasions and exposure to irritants may further impair the skin barrier and leads to refractory cases.

As with most types of dermatitis, topical corticosteroids or calcineurin inhibitors are the mainstay of medical management, particularly in low‐to‐moderate severity cases. A short course of oral prednisolone can be used for severe and recalcitrant cases. Prednisolone may additionally allow time for establishment of simple measures described above or be used as a bridging tool prior to commencing a steroid‐sparing agent.

An extended course of azathioprine resulted in an excellent response for the majority of patients in a study by Verma et al.^27^ in *Parthenium dermatitis*, at a dose of 300 mg once weekly. Methotrexate was an alternative option and has been used in once‐weekly doses of 15 mg.^28^ Both PUVA and narrowband UVB have been shown to have good effect in patients with severe Compositae dermatitis.^29^, ^30^ Lakshmi et al.^31^ have shown Cyclosporine to be effective in the acute phase at a dose of 2.5 mg/kg daily with a recommended treatment course of 4–8 weeks as a crisis intervention. The use of dupilumab in ACD is currently undergoing clinical trials and there are currently no reported cases of Compositae dermatitis treated with this agent.^32^

## 6 CONCLUSION

Although direct exposure from outdoor activities and gardening remains the most common cause of ACD to Compositae, exposure to personal products containing its extracts should be considered. Sensitisation to Compositae may lead to airborne contact dermatitis which is particularly difficult to manage and may be associated with the development of CAD. Identification and avoidance of Compositae remains the most crucial aspect of management in the face of limited treatment options.

## AUTHOR CONTRIBUTIONS

**Nisal Punchihewa:** Writing – original draft; formal analysis. **Amanda Palmer:** Data curation. **Rosemary Nixon:** Conceptualization; writing – review and editing.

## CONFLICT OF INTEREST

The authors declare no conflicts of interest.

## Data Availability

The data that support the findings of this study are available from the corresponding author upon reasonable request.
